# Knowledge, Awareness, and Willingness Toward the HPV Vaccine Among Medical Students at Qassim University: A Cross-Sectional Study

**DOI:** 10.3390/vaccines14060529

**Published:** 2026-06-15

**Authors:** Ghadah Alhetheli, Rifal Alhumaid, Shams Alajlan, Sheyam Alajlan, Lamia Alharbi, Lamis Allahim, Hala Ahmed Alrubah

**Affiliations:** 1 Department of Dermatology, College of Medicine, Qassim University, Buraydah 52571, Saudi Arabia; 2 College of Medicine, Qassim University, Buraydah 52571, Saudi Arabia; 431201405@qu.edu.sa (R.A.); 421201910@qu.edu.sa (S.A.); 431201551@qu.edu.sa (S.A.); 431201553@qu.edu.sa (L.A.); 431201475@qu.edu.sa (L.A.); 431201617@qu.edu.sa (H.A.A.)

**Keywords:** human papillomavirus, HPV, vaccines, cervical cancer, medical students, malignancies, awareness

## Abstract

**Background:** Human papillomavirus (HPV) causes nearly all cervical cancers and a growing share of HPV-related malignancies in both sexes, yet HPV vaccine knowledge and acceptance among medical students in Saudi Arabia, the next generation of clinicians shaping recommendations, remain poorly characterized across the full training continuum. **Methods:** We conducted a cross-sectional online survey of 300 medical students at the College of Medicine, Qassim University. A pre-validated questionnaire captured sociodemographic data, HPV knowledge (14 items), vaccine awareness, attitudes and behavioral intent (4 items), and barriers. Multivariable logistic regression assessed independent predictors of awareness, personal willingness, and intent to recommend the vaccine to family members and future patients. **Results:** A total of 91.7% of students had previously heard of HPV, and 79.3% had heard of the HPV vaccine. However, only 44.3% reached the predefined threshold for good knowledge, and 56.3% reported personal willingness to receive the vaccine. Willingness to recommend the vaccine to future patients was the most frequently endorsed intent (78.3%), followed by recommending it to a family member (73.3%), with male gender reported as the leading reason among decliners. After adjustment, each one-point increase in the knowledge score independently raised the adjusted odds of vaccine awareness (aOR 1.40, 95% CI 1.22 to 1.61), of recommending the vaccine to a future patient (aOR 1.28, 95% CI 1.13 to 1.45), of recommending it to a family member (aOR 1.19, 95% CI 1.06 to 1.33), and of personal willingness (aOR 1.16, 95% CI 1.04 to 1.29). Female gender was associated with higher odds of personal willingness (aOR 2.31, 95% CI 1.37 to 3.88), and senior training phase predicted vaccine awareness (aOR 2.71, 95% CI 1.33 to 5.52). **Conclusions:** Human papillomavirus vaccine knowledge independently predicted both awareness and behavioral intent among medical students at Qassim University. However, personal willingness to receive the vaccine lagged behind willingness to recommend it, particularly among male students. Embedding HPV prevention more explicitly into the medical curriculum, with particular emphasis on its relevance to male health, may help narrow this gap.

## 1. Introduction

Human papillomavirus (HPV) is the most prevalent sexually transmitted virus worldwide and the well-established causative agent of nearly all cases of cervical cancer, as well as a significant proportion of cancers of anal, vulvar, vaginal, penile, and oropharyngeal malignancies [1,2]. Based on GLOBOCAN 2022 estimates, cervical cancer remains a major cause of morbidity and mortality among female cancers worldwide, with over 600,000 new cases and 340,000 deaths annually, mostly in developing countries [3,4]. In the Eastern Mediterranean Region, cervical cancer continues to represent a predominant malignancy among women, with the burden varying in line with human development indicators across countries [5,6]. The increasing prevalence of HPV-associated disease in males, particularly the rise in HPV-related oropharyngeal cancer, has reshaped the discussion regarding HPV vaccines as cancer prevention tools [2].

Prophylactic HPV vaccines are highly efficacious when administered before exposure and have been shown to reduce the risk of invasive cervical cancer [7,8]. The WHO global strategy for the elimination of cervical cancer as a major public health problem aims for a 90% vaccination coverage rate in girls 15 years of age, together with wide coverage of screening and treatment. Studies suggest that, if attained, these targets could prevent millions of cervical cancer cases in the coming decades [9,10,11]. Recommendation of the vaccine by healthcare providers has emerged as one of the strongest predictors of HPV vaccine uptake and is therefore a key factor in achieving these targets [12,13,14].

Although Saudi Arabia has included the HPV vaccine in its national vaccination program, its uptake remains limited, and public awareness of HPV-related disease and vaccination is relatively low [15]. Across the region, research conducted in conservative Arab communities has consistently identified knowledge gaps, misconceptions, and cultural hesitancy toward diseases caused by sexually transmitted infections and toward vaccination programs [16,17,18]. While several studies in Saudi Arabia have assessed knowledge among female health college students and among students in early clinical years, few have provided a comprehensive picture of knowledge, attitudes, and willingness across all phases of medical training [19,20,21,22,23]. Medical students play an instrumental role in counseling and recommending HPV vaccination; assessing their preparedness for this role is therefore essential.

In Saudi Arabia, GLOBOCAN 2022 estimated 332 new cervical cancer cases, 164 deaths, and 1224 women living with a five-year cervical cancer diagnosis, indicating a preventable but continuing national burden [3]. The Saudi Ministry of Health describes HPV vaccination as a key cervical cancer prevention measure, with girls aged 9 to 14 years receiving two doses and girls and women aged 15 years or older receiving three doses [24]. Despite national availability, published Saudi studies continue to report low uptake, incomplete awareness of vaccine availability, and hesitancy related to perceived low personal risk, safety concerns, cultural sensitivity around sexually transmitted infections, and limited provider recommendation [15,20]. Medical students are therefore a strategic group because their knowledge, awareness, and willingness may influence their future vaccine counseling, recommendation practices, and the wider public acceptance of HPV vaccination.

This cross-sectional study was conducted at the College of Medicine, Qassim University, to assess the awareness of HPV-related diseases and knowledge, attitudes, and willingness to vaccinate or recommend vaccination. In addition, the influence of training phase, gender, source of information, and knowledge score on vaccination awareness, personal willingness, and willingness to recommend vaccination for family members and future patients was examined.

## 2. Methods

### 2.1. Study Design and Setting

A cross-sectional online survey was conducted among medical students enrolled at the College of Medicine, Qassim University (Al-Mulida branch), Saudi Arabia, between January 2026 and April 2026. This study aimed to assess HPV knowledge, vaccine awareness, attitudes, and willingness to vaccinate or recommend vaccination across the seven training cohorts. Reporting followed the STROBE statement.

The College of Medicine delivers a six-year undergraduate medical curriculum, followed by a one-year internship, and the training continuum was represented by seven cohorts (first through sixth year and the internship year). The finite target population comprised approximately 900 eligible medical students at the Al-Mulida branch. The completed STROBE checklist is provided as Supplementary Materials.

### 2.2. Participants and Sampling

The target population comprised all medical students enrolled at the College of Medicine, Qassim University, from the first preclinical year through to internship. A stratified systematic sampling procedure was used to recruit a representative sample across the seven training cohorts. Inclusion criteria were current enrolment in the College of Medicine, Qassim University, ability to understand and respond to the questionnaire in English, and provision of informed electronic consent. Participation was voluntary, anonymous, and confidential. The achieved sample of 300 participants was considered sufficient to provide stable proportion estimates within each cohort and to support multivariable logistic regression with up to ten predictors.

Stratified systematic sampling was chosen instead of simple random sampling to guarantee proportionate representation across the seven cohorts, which differ in size; within each stratum, students were selected at fixed intervals from the enrolment list. The required sample size was calculated in OpenEpi version 3 for a finite population of 900, assuming a 50% expected frequency, 5% absolute precision, a 95% confidence level, and a design effect of 1, which yielded a minimum of 270.

### 2.3. Study Instrument

The questionnaire was adapted from a previously validated instrument administered to medical students [25] and was modified for the Saudi context and current HPV guidance; the item on the principal female cancer asked about cancer in general and is therefore reported as cancer awareness rather than cervical cancer-specific knowledge. A pre-validated self-administered questionnaire was used, organized in five domains. Part 1 captured sociodemographic data. Part 2 assessed general HPV knowledge across nine items covering awareness, transmission, asymptomatic infection, sex distribution, sex-related risk profile, treatability, and recommended cervical-cytology frequency. A nested six-item module on non-sexual transmission routes was administered conditionally to respondents who did not endorse exclusively sexual transmission. Part 3 covered HPV vaccine knowledge across nine items (awareness, post-infection efficacy, indications for genital-warts patients, indications for boys, optimal timing, dose count, eligibility for adults aged 14 to 45 years, availability in Saudi Arabia, and the percentage of cervical cancers preventable by vaccination). Part 4 captured attitudes and behavioral intent across four items, with a structured barriers module presented to respondents who declined any of the first three intent items. Part 5 captured the self-reported source of HPV vaccine information.

### 2.4. Data Recoding and Scoring

Item-level knowledge responses were dichotomized against a reference key derived from current CDC, WHO, and Saudi Ministry of Health guidance, with respondents endorsing the ‘I do not know’ option coded as incorrect. A composite knowledge score was constructed as the sum of fourteen items (1 = correct, 0 = incorrect). Awareness of the HPV vaccine, which served as a dependent variable, and the six transmission-route items, which were administered conditionally on a previous skip pattern, were excluded from the score. The score was dichotomized at the 60% threshold (≥9 of 14 items correct) into good and poor knowledge categories. A composite attitude/intent score (range 0 to 4) was constructed by summing four binary endorsements; a score of ≥3 was classified as positive intent.

### 2.5. Validity and Reliability

Content validity was supported by adapting items from previously validated regional instruments and by reference to current public health guidance for the answer key. Face validity was confirmed during questionnaire pre-testing. Internal consistency of the fourteen-item knowledge composite was acceptable (Cronbach α = 0.685, mean inter-item r = 0.14), and consistency of the four-item attitude/intent composite was good (Cronbach α = 0.765, mean inter-item r = 0.46). Item-rest correlations for the knowledge composite ranged from +0.04 to +0.46; only one item (vaccine not protective if already infected) fell below the conventional threshold of +0.20, and removing it would have raised α to 0.705. Item-rest correlations for the attitude/intent composite were uniformly strong (range +0.45 to +0.71).

### 2.6. Statistical Analysis

Categorical variables were summarized as n/N (%); for binary items, only the ‘Yes’ category is reported. Because the knowledge and attitude/intent scores were bounded discrete variables, they were summarized as the median (interquartile range, IQR) and compared between groups using the Wilcoxon rank-sum test for two-group comparisons and the Kruskal–Wallis test for comparisons of three or more groups, with Dunn post hoc tests (Bonferroni-adjusted) where the omnibus test was significant; inspection of the score distributions confirmed non-normality and supported the use of nonparametric methods. Year of study was treated as a seven-level factor in the univariate analysis and collapsed into a binary training phase (Junior, first to third year; Senior, fourth year to internship) for the regression models. For each outcome, crude logistic-regression models were fitted for every candidate predictor, followed by an adjusted multivariable model containing training phase, gender, source of information, and the continuous knowledge score (except in the model for good knowledge, in which the score defined the outcome) as a priori predictors. Models were fitted for good knowledge, positive attitude/intent, personal willingness to take the vaccine, willingness to recommend the vaccine to a family member, willingness to recommend it to future patients, and awareness of the HPV vaccine. Results are reported as crude and adjusted odds ratios (OR and aOR) with 95% confidence intervals (CI). Model adequacy was assessed using event counts, variance inflation factors, calibration by the Hosmer–Lemeshow test, and discrimination by the area under the receiver operating characteristic curve (AUC). All analyses were performed in R version 4.3.2 (R Foundation for Statistical Computing, Vienna, Austria); statistical significance was set at two-tailed *p* < 0.05.

### 2.7. Ethical Considerations

The study protocol was reviewed and approved by the Institutional Review Board of Qassim University on 7 January 2026, with approval reference number ERC QU 26-20-08. The research was carried out in accordance with the Declaration of Helsinki’s rules. All participants provided informed electronic consent before completing the questionnaire. Data were anonymized at the source and stored on a password-protected server accessible only to the research team.

## 3. Results

### 3.1. Sociodemographic Characteristics

A total of 300 medical students completed the questionnaire (Table 1). The mean age was 22.10 ± 2.14 years, with the majority (153/300, 51.0%) aged 21 to 23 years. Male students predominated (170/300, 56.7%). The five most represented years were fifth year (64/300, 21.3%), fourth year (50/300, 16.7%), sixth year (48/300, 16.0%), third year (46/300, 15.3%), and second year (39/300, 13.0%); first-year students (21/300, 7.0%) and interns (32/300, 10.7%) were the smallest cohorts. Medical education was the leading reported source of HPV vaccine information (230/300, 76.7%), followed by self-reading (41/300, 13.7%) and social media or interpersonal sources (29/300, 9.7%).

### 3.2. HPV Knowledge Items

**Figure 1:**
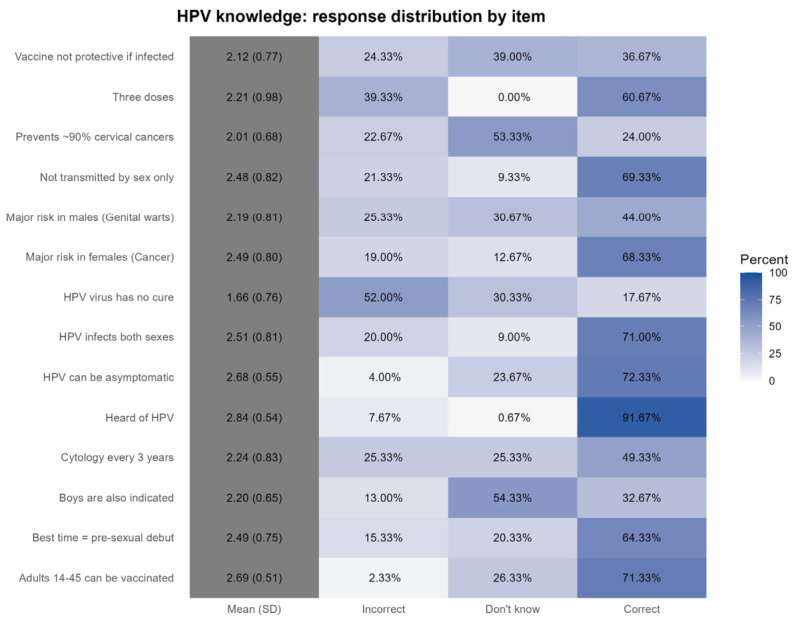
HPV knowledge: response distribution by item. Likert-style heatmap shows the percentage of respondents giving each response category (Incorrect, I do not know, Correct) for the fourteen knowledge items.

As shown in Table 2, item-level performance varied widely across the fourteen knowledge items. Awareness of HPV reached 91.7% across the cohort. The next-best-known items were the eligibility of adults aged 14 to 45 years for vaccination (71.3%), recognition that HPV affects both sexes (71.0%), and that HPV can be asymptomatic (72.3%). The lowest-scoring items were the recognition that HPV virus has no cure (17.7%), the proportion of cervical cancers preventable by vaccination (24.0% identified the ~90% benchmark), and the indication for vaccinating boys (32.7%). Three items had a clear plurality of I-do-not-know responses (vaccine not protective if infected, indication for boys, and the major risk in males), pointing to specific knowledge gaps rather than active misinformation. Among respondents who endorsed at least one non-sexual route of transmission, mother-to-fetus transmission was correctly recognized by 68.5%, skin-to-mucosa contact by 67.3%, and skin-to-skin contact by 50.8%; correct rejection of contaminated water as a transmission route was achieved by 46.7% and contaminated medical equipment by only 16.0%. The corresponding Likert response distribution is visualized in Figure 1.

### 3.3. Vaccine Awareness, Attitudes, and Intent Items

**Figure 2:**
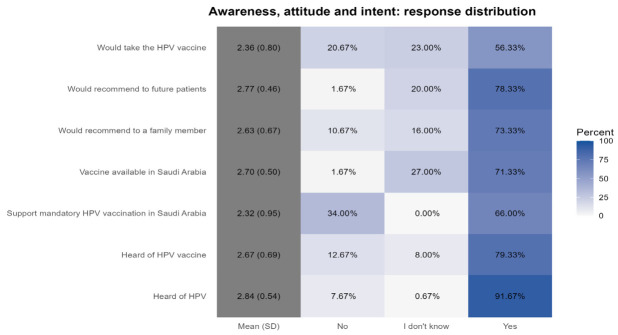
Vaccine awareness, attitude and intent: response distribution. Heatmap shows the percentage of respondents endorsing each response category (No, I do not know, Yes) for the seven items.

Awareness of the HPV vaccine reached 238/300 (79.3%) across the cohort, and awareness that the vaccine is available in Saudi Arabia reached 214/300 (71.3%) (Table 3). Across the four behavioral-intent items, willingness to recommend the vaccine to future patients was the most frequently endorsed (235/300, 78.3%), followed by willingness to recommend it to a family member (220/300, 73.3%), support for mandatory HPV vaccination (198/300, 66.0%), and personal willingness to take the vaccine (169/300, 56.3%). Personal willingness was the only item for which ‘No’ and ‘I-do-not-know’ responses together exceeded 40%, indicating that the gap between recommending the vaccine to others and personally accepting it is the primary source of hesitancy within the cohort. The full response distribution for vaccine awareness, attitude, and intent items is visualized in Figure 2.

### 3.4. Barriers to Vaccination

**Figure 3:**
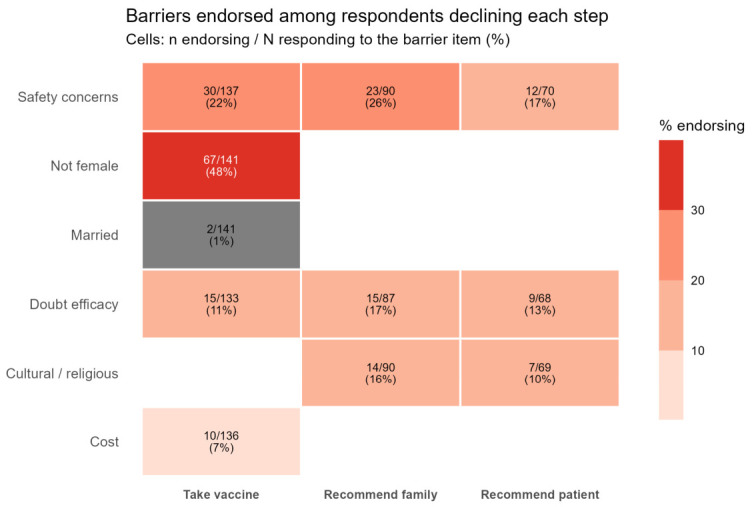
Barriers to HPV vaccination. Heatmap shows the percentage of respondents declining each step (taking the vaccine, recommending it to family, recommending it to patients) who endorsed each reason.

Among the 131 respondents who declined to take the vaccine themselves, the most frequently endorsed barrier was not being female (47.5% of decliners), followed by safety concerns (21.9%), doubts about protective efficacy (11.3%), cost (7.4%), and being married (1.4%) (Table 4). Among the 90 respondents reluctant to recommend the vaccine to a family member, safety concerns were endorsed by 25.6%, doubts about efficacy were endorsed by 17.2%, and cultural or religious concerns were endorsed by 15.6%. Endorsement was lower across all reasons among the 70 respondents reluctant to recommend the vaccine to future patients (safety 17.1%, efficacy 13.2%, cultural or religious 10.1%). The distribution of barriers across all three decline steps is visualized in Figure 3.

### 3.5. Composite Scores and Univariate Associations

The median knowledge score was 8 of 14 (IQR 6 to 10), and 133/300 respondents (44.3%) met the threshold for good knowledge (≥9 of 14 items correct). The median attitude/intent score was 3 of 4 (IQR 2 to 4), with 199/300 (66.3%) classified as having positive intent. Median scores by predictor and the corresponding nonparametric tests are presented in Table 5. Knowledge scores rose across the training continuum, from a median of 4 (IQR 1 to 6) in first-year students to 10 (IQR 8 to 11) in sixth-year students, and differed significantly by year of study, training phase, gender, age group, and source of information (all *p* < 0.05). The age-group gradient was monotonic (median 6, 8, and 9 for the youngest, middle, and oldest groups; Kruskal–Wallis *p* < 0.001), and Dunn post hoc testing confirmed that each age group differed significantly from the others. For the attitude/intent score, significant differences were observed by training phase, gender, and source of information, whereas year of study (*p* = 0.052) and age group (*p* = 0.080) did not reach significance; the largest contrast was by source of information, with students relying on medical education scoring higher than those relying on other or self-directed sources (Kruskal–Wallis *p* < 0.001).

### 3.6. Correlation Between Knowledge and Acceptance

**Figure 4:**
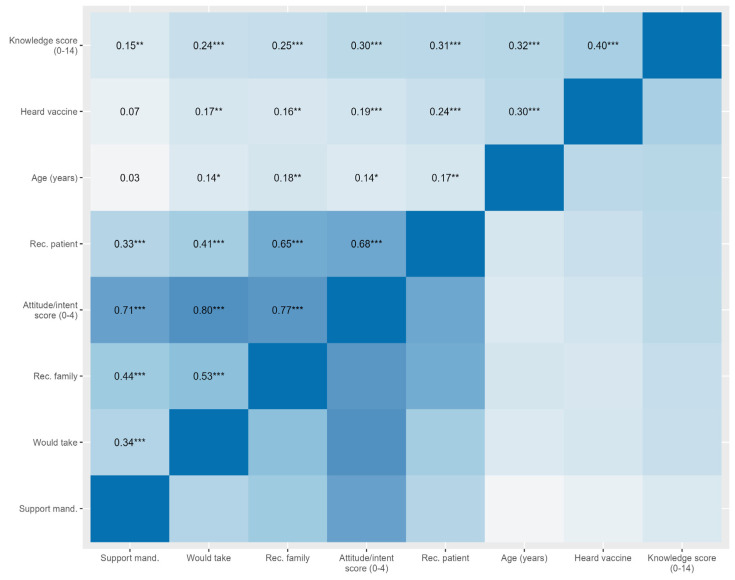
Correlation matrix between the composite knowledge score, the four behavioral-intent items, the composite attitude/intent score, awareness of the HPV vaccine, and age. Cells show the Spearman rank correlation coefficient. Asterisks: * *p* < 0.05, ** *p* < 0.01, *** *p* < 0.001.

The Spearman rank-correlation matrix between the composite knowledge score, the four behavioral-intent items, the composite attitude/intent score, awareness of the HPV vaccine, and age is shown in Figure 4. Knowledge correlated positively and significantly with every acceptance and intent measure. The strongest correlation was with awareness of the HPV vaccine (ρ = +0.39, *p* < 0.001), followed by willingness to recommend the vaccine to future patients (ρ = +0.31, *p* < 0.001), the composite attitude/intent score (ρ = +0.30, *p* < 0.001), willingness to recommend the vaccine to a family member (ρ = +0.25, *p* < 0.001), and personal willingness to take the vaccine (ρ = +0.24, *p* < 0.001). The correlation with support for mandatory vaccination was weaker but still significant (ρ = +0.15, *p* = 0.007). Knowledge also correlated positively with age (ρ = +0.32, *p* < 0.001), and the attitude/intent score correlated weakly but significantly with age (ρ = +0.14, *p* = 0.016). Within the acceptance items themselves, the strongest pairwise correlation was between recommending to family and recommending to patients (ρ = +0.65), reflecting a single underlying recommendation tendency.

### 3.7. Multivariable Predictors of Intent and Awareness

**Figure 5:**
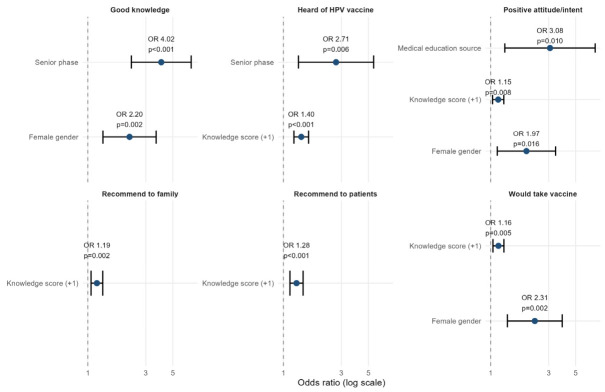
Adjusted odds ratios (95% CI) for the statistically significant predictors in the multivariable logistic-regression models. Estimates are plotted on a logarithmic scale, and the dashed vertical line denotes the null value (OR = 1).

Crude and adjusted logistic-regression results for all six outcomes are summarized in Table 6. After adjustment for training phase, gender, source of information, and the knowledge score, the knowledge score was independently associated with every outcome in which it was entered as a predictor. For each one-point increase, the adjusted odds rose by 40% for vaccine awareness (aOR 1.40, 95% CI 1.22 to 1.61, *p* < 0.001), 28% for recommending the vaccine to a future patient (aOR 1.28, 95% CI 1.13 to 1.45, *p* < 0.001), 19% for recommending it to a family member (aOR 1.19, 95% CI 1.06 to 1.33, *p* = 0.002), 16% for personal willingness (aOR 1.16, 95% CI 1.04 to 1.29, *p* = 0.005), and 15% for positive attitude/intent (aOR 1.15, 95% CI 1.04 to 1.28, *p* = 0.008). Female students were more likely to report personal willingness (aOR 2.31, 95% CI 1.37 to 3.88, *p* = 0.002), positive attitude/intent (aOR 1.97, 95% CI 1.13 to 3.42, *p* = 0.016), and good knowledge (aOR 2.20, 95% CI 1.33 to 3.65, *p* = 0.002). Senior training phase independently predicted vaccine awareness (aOR 2.71, 95% CI 1.33 to 5.52, *p* = 0.006) and good knowledge (aOR 4.02, 95% CI 2.28 to 7.08, *p* < 0.001), and a medical-education information source predicted positive attitude/intent (aOR 3.08, 95% CI 1.31 to 7.24, *p* = 0.010). All adjusted models showed acceptable discrimination (AUC 0.70 to 0.81), adequate calibration (Hosmer–Lemeshow *p* > 0.15), and low collinearity (maximum VIF < 1.2) Figure 5.

## 4. Discussion

This cross-sectional study assessed the awareness, knowledge, and behavioral intent regarding HPV across the various training phases at the College of Medicine, Qassim University. The results reveal relatively high HPV awareness, inconsistent item-level knowledge, greater willingness to recommend the vaccine to others than to receive it personally, and a statistically significant independent association between knowledge scores and all four vaccination behavior indicators after adjustment for training phase, gender, and information source. To our knowledge, no other Saudi study has simultaneously profiled medical students’ knowledge and acceptance of the HPV vaccine across the full spectrum from the preclinical to the internship years or examined the four behavioral outcomes within a single model.

Regarding knowledge, the results from the present study are consistent with findings from Saudi Arabia and the wider Gulf region, showing that medical students tend to be aware of HPV and its link to cervical cancer, although their understanding of vaccination indications, age eligibility, vaccine treatability, and the proportion of cervical cancer cases preventable by the vaccine remains incomplete [19,21,22,23]. As three questionnaire items received more “I do not know” responses than correct answers in our cohort, the gap appears to reflect limited knowledge rather than misinformation. Specifically, there is a notable discrepancy between recognition that HPV causes cervical cancer (93.7%) and recognition that the vaccine prevents 90% of such cases (16%). Indeed, other research has shown that pharmacy and nursing students in Saudi Arabia also underestimate the efficacy of the HPV vaccine despite being aware of its causative role in cervical cancer [26,27,28].

Building on the knowledge profile, we found that the overall score was positively associated with every acceptance and recommendation measure used and independently predicted HPV vaccine awareness, personal willingness, and willingness to recommend the vaccine to family members or future patients after adjustment for potential confounders. The strongest association was observed with HPV vaccine awareness, followed by willingness to recommend the vaccine to future patients, suggesting that knowledge correlates more closely with baseline awareness than with behavioral intent. These findings mirror regional reports of positive associations between HPV knowledge and acceptance or recommendation intent among medical and health science students in Jordan, Qatar, and Lebanon [17,25,29]. Although our design does not permit causal inference, the findings nonetheless support the framing of knowledge as a modifiable cognitive determinant of vaccine acceptance and recommendation, as specified in the WHO Vaccine Hesitancy Determinants Matrix [30].

More notably, the present research reveals a substantial gap between willingness to recommend the vaccine to others and personal willingness to receive it. While 78% of participants reported willingness to recommend the vaccine to future patients, only 56% indicated personal willingness to take it, as the primary barrier to personal uptake was male gender. This finding aligns with regional research showing that personal willingness among medical students is disproportionately low and that perceptions of irrelevance are particularly common among men [20,31,32]. The finding that female gender was a statistically significant predictor of personal willingness without predicting recommendation is fully consistent with the historical framing of the HPV vaccine as a women-only vaccine, whereas male students appear to underestimate HPV-related risks. A targeted message addressing this issue should be incorporated into the curriculum.

The gap between recommendation intent and personal willingness may be driven by several hesitancy factors. These include the historical framing of HPV vaccination as a female-focused cervical cancer intervention, the perception among male students that vaccination is personally irrelevant, uncertainty about vaccine safety or efficacy, cost and access concerns, and cultural sensitivity surrounding a sexually transmitted infection. Educational messaging should therefore frame HPV vaccination as gender-relevant cancer prevention and should explicitly address male HPV-related disease and vaccine safety.

On further examination of the predictors, we found that senior training phase independently predicted HPV vaccine awareness but showed no statistically significant association with any other variable. The source of information was independently associated with attitude and intent, but the effect attenuated after the introduction of knowledge as an independent variable, suggesting that knowledge was primarily responsible for the association. Although our findings echo the regional and multinational literature linking the source of information to health behavior [31,33], knowledge might be the mediating variable underlying that link. Therefore, curricular content, rather than the source of information per se, should be the primary target for intervention.

Based on these findings, several educational directions may be considered. First, medical curricula should emphasize HPV vaccine prevention before clinical exposure, highlighting the importance of vaccinating boys and the role of the vaccine in HPV-related cancers other than cervical cancer. This would enable medical students to better understand the HPV vaccination targets set by the WHO elimination strategy [9,10,14]. Second, training in effective communication skills aimed at promoting vaccine recommendation to individuals of both sexes is also important. As research indicates that physicians’ recommendations are major determinants of vaccine uptake, such training would be beneficial [12,13]. Short-term educational interventions in schools and universities have already demonstrated effectiveness in promoting vaccination and recommendation intention [34,35]; however, such education is likely to be most effective when combined with easy on-campus access to vaccination.

Building on the item-level findings, the lowest-scoring knowledge items, namely recognition that HPV has no curative treatment (17.7% correct), the proportion of cervical cancers preventable by vaccination (24.0%), and the indication for vaccinating boys (32.7%), represent priority targets for curricular reinforcement. The persistence of these specific gaps in a population that will soon counsel patients on HPV prevention is concerning, particularly when set against the WHO 2030 cervical cancer elimination targets, which depend on broad provider literacy regarding vaccine indications and effectiveness [36]. Comparable misconceptions about treatability and male indications have been documented across Saudi medical schools and other Gulf institutions, suggesting that the gap reflects systematic underexposure rather than student-level effort [20,22]. Embedding short, structured modules on HPV epidemiology, the gender-neutral vaccine schedule, and post-infection vaccination caveats into preclinical years, with reinforcement during clinical rotations, is therefore proposed as a practical remediation strategy.

The 79.3% vaccine awareness reported in the present cohort is broadly consistent with figures from comparable Saudi medical-student populations [20,21] and slightly higher than awareness levels reported in earlier multi-country Arab samples, which ranged between 35% and 65% [16]. Despite this relatively favorable awareness, only 71.3% of participants were aware that the vaccine is available within Saudi Arabia, suggesting that information about local accessibility lags behind general knowledge of the vaccine. Plausible drivers include limited curricular coverage of the national immunization schedule, the absence of a structured male catch-up program and the historical framing of HPV as a female-only concern in regional health communication [17,19]. Closing this awareness–availability gap likely requires the integration of national program details into preclinical curricula and the inclusion of HPV vaccination among the routine counseling competencies assessed during clinical training.

This study has several strengths. It recruited participants across the full medical-training continuum, from the preclinical years to internship; included both male and female students; assessed awareness, knowledge, personal willingness, recommendation intent, and barriers within a single survey; and used multivariable modeling with crude and adjusted estimates and formal model diagnostics. Several limitations also warrant mention. The cross-sectional design precludes causal inference, and recruitment from a single university limits generalizability beyond Qassim University, particularly given the systematically sampled, non-probability nature of the achieved sample. Self-report and the survey format may introduce social-desirability and self-selection biases; these risks were minimized by anonymous electronic data collection, voluntary participation, standardized questionnaire wording, and the absence of identifying data in the analysis file. The fourteen-item knowledge composite had acceptable internal consistency (alpha = 0.685), which was mitigated by a three-agency consensus answer key, a sensitivity analysis on the two contentious items, and reliance on the highly reliable four-item attitude/intent composite. Finally, most participants were older than the primary target age for routine adolescent HPV vaccination, so their personal willingness should be interpreted as adult catch-up or hypothetical acceptance rather than routine adolescent-program uptake.

In conclusion, this cross-sectional study found that both awareness and willingness to recommend the HPV vaccine increase with advanced training phase and higher knowledge scores. Conversely, personal willingness was less influenced by academic progression, correlating instead with gender-related perceptions. Greater emphasis on vaccine prevention in medical curricula, with particular attention to indications in males and a cancer-prevention framing, would be beneficial. Specific item-level gaps in the recognition that HPV has no curative treatment, in the proportion of cervical cancers preventable by vaccination, and in the indication for vaccinating boys represent immediate priorities for curricular reinforcement. Future work should evaluate the impact of structured pre-clinical HPV modules through longitudinal pre–post designs, examine the qualitative drivers of male vaccine hesitancy in this cohort, and extend the present survey to a multi-center Saudi medical-student sample to support generalizability.

To translate these findings into practice, medical-student interventions should include structured preclinical HPV modules, explicit teaching of the Saudi vaccination schedule, gender-neutral cancer-prevention framing that addresses HPV-related disease in both sexes, communication training for vaccine counseling, myth-correction content on vaccine safety and efficacy, and practical linkage to on-campus or primary-care vaccination services where feasible.

## Figures and Tables

**Table 1:** Sociodemographic characteristics of the sample (N = 300).

Variable	Total (n = 300)
Age (years), mean ± SD	22.10 ± 2.14
Age group, n (%)	
≤20 years	71 (23.7)
21 to 23 years	153 (51.0)
≥24 years	75 (25.0)
Gender, n (%)	
Male	170 (56.7)
Female	130 (43.3)
Year of study, n (%)	
1st	21 (7.0)
2nd	39 (13.0)
3rd	46 (15.3)
4th	50 (16.7)
5th	64 (21.3)
6th	48 (16.0)
Intern	32 (10.7)
Source of HPV vaccine information, n (%)	
Medical education	230 (76.7)
Self-reading	41 (13.7)
Social media/family/friends	29 (9.7)

Continuous variables are reported as mean ± SD. Categorical variables are reported as n (%).

**Table 2:** Item-level knowledge correctness (N = 300).

Knowledge Item (Correct Response)	n Correct	% Correct
Have you heard of HPV? (Yes)	275	91.7
Who does HPV infect? (Both sexes)	213	71.0
Can HPV be asymptomatic? (Yes)	217	72.3
Is HPV transmitted only by sex? (No)	208	69.3
Major risk in females (Cancer)	205	68.3
Major risk in males (Genital warts)	132	44.0
Can HPV be cured? (No, virus has no cure)	53	17.7
Cytology frequency (Every 3 years)	148	49.3
Vaccine protective if already infected? (No)	110	36.7
Are boys indicated for vaccination? (Yes)	98	32.7
Best time to vaccinate (Children 9–13/pre-sexual debut)	193	64.3
Number of doses (Three)	182	60.7
Can adults 14–45 be vaccinated? (Yes)	214	71.3
% of cervical cancers preventable by vaccination (~90%)	72	24.0

Cells show the number and percentage of respondents giving the correct response, which is shown in parentheses next to each item.

**Table 3:** Vaccine awareness, attitude, and intent items (N = 300).

Attitude/Intent Item (Yes Response)	n	%
Have you heard of the HPV vaccine? (Yes)	238	79.3
Is the vaccine available in Saudi Arabia? (Yes)	214	71.3
Would you take the HPV vaccine?	169	56.3
Would you recommend the vaccine to a family member?	220	73.3
Would you recommend the vaccine to your future patients?	235	78.3
Do you support mandatory HPV vaccination in Saudi Arabia?	198	66.0

Cells show the number and percentage of respondents endorsing the Yes option.

**Table 4:** Barriers to vaccination endorsed by respondents who declined each step.

Reason for Declining	Would Not Take (n = 131)	Would Not Recommend to Family (n = 90)	Would Not Recommend to Patient (n = 70)
Not being female	67 (47.5)	—	—
Safety concerns	30 (21.9)	23 (25.6)	12 (17.1)
Doubts about protective efficacy	15 (11.3)	15 (17.2)	9 (13.2)
Cultural or religious concerns	—	14 (15.6)	7 (10.1)
Cost of the vaccine	10 (7.4)	—	—
Already married	2 (1.4)	—	—

Cells show n (% of decliners endorsing the reason). Em dashes indicate the reason was not asked for that step.

**Table 5:** Knowledge and attitude/intent scores by sociodemographic and academic predictor.

Variable	Level	Knowledge Score, Median (IQR)	Knowledge *p*	Attitude/Intent Score, Median (IQR)	Attitude/Intent *p*
Year of study	1st year	4 (1–6)	<0.001	1 (1–3)	0.052
	2nd year	6 (5–8)		3 (2–4)	
	3rd year	7 (5–9)		3 (1–4)	
	4th year	8 (7–10)		4 (1–4)	
	5th year	9 (7–10)		3 (2–4)	
	6th year	10 (8–11)		4 (2–4)	
	Internship	9 (8–10)		3 (2–4)	
Training phase	Junior (1st–3rd)	6 (4–8)	<0.001	3 (1–4)	0.045
	Senior (4th–Intern)	9 (7–10)		3 (2–4)	
Gender	Male	8 (6–9)	0.004	3 (2–4)	0.001
	Female	9 (7–10)		4 (2–4)	
Age group	≤20 years	6 (4–8)	<0.001	3 (1–4)	0.080
	21–23 years	8 (6–10)		3 (2–4)	
	≥24 years	9 (7–10)		3 (2–4)	
Source of information	Other	7 (5–8)	<0.001	2 (1–3)	<0.001
	Self-reading	7 (2–10)		1 (0–4)	
	Medical education	8 (7–10)		3 (2–4)	

Data are summarized as median (IQR), with nonparametric tests. *p*-values are from the Wilcoxon rank-sum test (two groups) or the Kruskal–Wallis test (three or more groups). Where the Kruskal–Wallis omnibus test was significant, Dunn post hoc testing was applied: for knowledge, junior and younger groups differed significantly from senior and older groups, and a medical-education source differed from other and self-reading sources; for attitude/intent, a medical-education source differed from other and self-reading sources.

**Table 6:** Crude and adjusted logistic regression of vaccine-related outcomes.

Outcome	Predictor	Crude OR (95% CI), *p*	Adjusted OR (95% CI), *p*
Good knowledge	Senior phase (vs. Junior)	3.78 (2.24–6.40), *p* < 0.001	4.02 (2.28–7.08), *p* < 0.001
	Female (vs. Male)	1.77 (1.12–2.81), *p* = 0.015	2.20 (1.33–3.65), *p* = 0.002
	Self-reading (vs. Other)	1.81 (0.63–5.24), *p* = 0.272	1.96 (0.63–6.11), *p* = 0.243
	Medical education (vs. Other)	2.93 (1.21–7.13), *p* = 0.018	2.20 (0.85–5.71), *p* = 0.104
Positive attitude/intent	Senior phase (vs. Junior)	1.71 (1.04–2.80), *p* = 0.034	0.99 (0.54–1.80), *p* = 0.970
	Female (vs. Male)	1.96 (1.19–3.23), *p* = 0.008	1.97 (1.13–3.42), *p* = 0.016
	Self-reading (vs. Other)	1.11 (0.42–2.90), *p* = 0.834	0.96 (0.35–2.65), *p* = 0.940
	Medical education (vs. Other)	3.92 (1.77–8.69), *p* < 0.001	3.08 (1.31–7.24), *p* = 0.010
	Knowledge score (per point)	1.23 (1.12–1.35), *p* < 0.001	1.15 (1.04–1.28), *p* = 0.008
Would take vaccine	Senior phase (vs. Junior)	1.58 (0.98–2.54), *p* = 0.061	1.00 (0.56–1.77), *p* = 0.995
	Female (vs. Male)	2.30 (1.43–3.70), *p* < 0.001	2.31 (1.37–3.88), *p* = 0.002
	Self-reading (vs. Other)	0.85 (0.32–2.28), *p* = 0.745	0.70 (0.24–2.00), *p* = 0.502
	Medical education (vs. Other)	2.74 (1.24–6.08), *p* = 0.013	2.14 (0.90–5.07), *p* = 0.085
	Knowledge score (per point)	1.23 (1.12–1.34), *p* < 0.001	1.16 (1.04–1.29), *p* = 0.005
Recommend to family	Senior phase (vs. Junior)	1.75 (1.04–2.96), *p* = 0.036	0.93 (0.49–1.75), *p* = 0.823
	Female (vs. Male)	1.39 (0.82–2.35), *p* = 0.220	1.28 (0.72–2.27), *p* = 0.404
	Self-reading (vs. Other)	0.74 (0.28–1.94), *p* = 0.541	0.65 (0.24–1.77), *p* = 0.398
	Medical education (vs. Other)	2.68 (1.20–5.98), *p* = 0.016	1.87 (0.79–4.44), *p* = 0.156
	Knowledge score (per point)	1.24 (1.13–1.36), *p* < 0.001	1.19 (1.06–1.33), *p* = 0.002
Recommend to patients	Senior phase (vs. Junior)	2.48 (1.42–4.35), *p* = 0.001	1.09 (0.54–2.17), *p* = 0.812
	Female (vs. Male)	1.40 (0.80–2.47), *p* = 0.240	1.27 (0.66–2.43), *p* = 0.470
	Self-reading (vs. Other)	0.55 (0.21–1.47), *p* = 0.236	0.44 (0.15–1.29), *p* = 0.134
	Medical education (vs. Other)	2.93 (1.26–6.83), *p* = 0.013	1.64 (0.64–4.21), *p* = 0.302
	Knowledge score (per point)	1.36 (1.22–1.51), *p* < 0.001	1.28 (1.13–1.45), *p* < 0.001
Heard of HPV vaccine	Senior phase (vs. Junior)	5.20 (2.86–9.45), *p* < 0.001	2.71 (1.33–5.52), *p* = 0.006
	Female (vs. Male)	1.80 (1.00–3.26), *p* = 0.050	1.71 (0.85–3.46), *p* = 0.134
	Self-reading (vs. Other)	1.36 (0.51–3.63), *p* = 0.538	1.42 (0.42–4.77), *p* = 0.574
	Medical education (vs. Other)	3.80 (1.68–8.64), *p* = 0.001	1.59 (0.58–4.31), *p* = 0.366
	Knowledge score (per point)	1.54 (1.36–1.74), *p* < 0.001	1.40 (1.22–1.61), *p* < 0.001

Cells show the odds ratio (95% CI) and *p*-value.

## Data Availability

The data that support the findings of this study are available from the corresponding author upon reasonable request, subject to the data-sharing terms approved by the Qassim University Institutional Review Board.

## Supplementary Materials

The following supporting information can be downloaded at: https://www.mdpi.com/article/10.3390/vaccines14060529/s1, STROBE checklist [37].
