# Supplementary material for: Knowledge, Awareness, and Willingness Toward the HPV Vaccine Among Medical Students at Qassim University: A Cross-Sectional Study
Source: Vaccines (Basel). 2026 Jun 15;14(6):529. doi: 10.3390/vaccines14060529 (PMC13307675; doi:10.3390/vaccines14060529)
Supplement: Supplementary file 1 [file vaccines-14-00529-s001.zip › vaccines-4339861-supplementary.pdf]

Supplementary Materials:

STROBE checklist：
von Elm E, Altman DG, Egger M, Pocock SJ, Gøtzsche PC, Vandenbroucke JP; STROBE Initiative. J Clin Epidemiol. 2008;61(4):344–9. PMID: 18313558
